# Multipurpose HTS Coagulation Analysis: Assay Development and Assessment of Coagulopathic Snake Venoms

**DOI:** 10.3390/toxins9120382

**Published:** 2017-11-25

**Authors:** Kristina B. M. Still, Randjana S. S. Nandlal, Julien Slagboom, Govert W. Somsen, Nicholas R. Casewell, Jeroen Kool

**Affiliations:** 1 Division of BioAnalytical Chemistry, Department of Chemistry and Pharmaceutical Sciences, Faculty of Sciences, Vrije Universiteit Amsterdam, De Boelelaan 1085, 1081 HV Amsterdam, The Netherlands; kristina.still90@gmail.com (K.B.M.S.); r.s.s.nandlal@student.vu.nl (R.S.S.N.); j.slagboom@student.vu.nl (J.S.); g.w.somsen@vu.nl (G.W.S.); 2 Alistair Reid Venom Research Unit, Parasitology Department, Liverpool School of Tropical Medicine, Liverpool L3 5QA, UK; nicholas.casewell@lstmed.ac.uk; 3 Research Centre for Drugs and Diagnostics, Liverpool School of Tropical Medicine, Liverpool L3 5QA, UK

**Keywords:** snake venoms, blood coagulation, coagulation bioassay, high-throughput screening, mass spectrometry, liquid chromatography

## Abstract

Coagulation assays currently employed are often low throughput, require specialized equipment and/or require large blood/plasma samples. This study describes the development, optimization and early application of a generic low-volume and high-throughput screening (HTS) assay for coagulation activity. The assay is a time-course spectrophotometric measurement which kinetically measures the clotting profile of bovine or human plasma incubated with Ca^2+^ and a test compound. The HTS assay can be a valuable new tool for coagulation diagnostics in hospitals, for research in coagulation disorders, for drug discovery and for venom research. A major effect following envenomation by many venomous snakes is perturbation of blood coagulation caused by haemotoxic compounds present in the venom. These compounds, such as anticoagulants, are potential leads in drug discovery for cardiovascular diseases. The assay was implemented in an integrated analytical approach consisting of reversed-phase liquid chromatography (LC) for separation of crude venom components in combination with parallel post-column coagulation screening and mass spectrometry (MS). The approach was applied for the rapid assessment and identification of profiles of haemotoxic compounds in snake venoms. Procoagulant and anticoagulant activities were correlated with accurate masses from the parallel MS measurements, facilitating the detection of peptides showing strong anticoagulant activity.

## 1. Introduction

Blood coagulation, the process of blood solidifying into a gel-like fibrinous clot, is a highly complex and vital mechanism in vertebrates. Blood coagulation involves various proteases, inhibitors and counteracting proteases, with the process initiated upon damage to the inner surface of the blood vessel, stimulating activation of the blood clotting cascade, and ultimately resulting in fibrinogen being transformed into fibrin-based gelatinous clots [1,2]. Deregulation of these processes can lead to bleeding disorders, resulting in either prolonged/excessive bleeding or unwanted blood coagulation, for example, thrombosis, which can be life threatening.

The effects of blood coagulation deregulation are among the main causes of human death in the developed world, primarily in the form of stroke and myocardial infarction [3,4,5]. In addition, the consistent increase in cardiovascular diseases [3] means blood disorder research and the tests that monitor blood coagulation are crucial aspects in clinical diagnostics. Existing diagnostic tests include complete blood counts, prothrombin time (PT), activated partial thromboplastin time (aPTT), fibrinogen level, platelet count, thrombin time (TT), bleeding time and so on. These tests are often laborious, require specialized equipment and/or may require large sample volumes of blood or plasma. As an example, the PT, aPTT and TT are onerous, requiring the addition of special reagents to initiate the test [6,7], and are often low-throughput. While most of these assays are, or can be, automated, with specialized robotic equipment (e.g., Stago equipment [8]) they are not easily implemented in every lab. While the existing blood coagulation tests are indispensable for the diagnostics workflow due to their specificity in relation to determining the causes of coagulation disorders, a simple high-throughput assaying method which requires a low sample volume for the screening and detection of coagulation disruption would undoubtedly be valuable.

In addition to their utility in clinical diagnostics, rapid high-throughput coagulation assays would also be valuable tools for screening new compounds with anti- and procoagulation effects. These compounds could include small molecular hits in drug discovery pipelines or bioactive peptides and/or proteins as biopharmaceutical candidates. The current need to replace warfarin, an anticoagulant most commonly used in the treatment and prevention of venous thrombosis and thromboembolic complications associated with atrial fibrillation, is a prime example [9,10]. Warfarin is difficult to dose and requires close monitoring of blood levels, the target international normalized ratio (INR) in the body, requiring daily INR monitoring in hospital until it has reached a stable level. In addition, warfarin is known to have drug–drug and drug–food interactions and possible severe side effects to patients upon administration, such as high increases in INR and bleeding complications upon deficiency of factor VII, or occurrence of hypercoagulable state upon protein C deficiency, all resulting in prolonged hospitalization times [9,10,11]. For this reason, natural products such as plant extracts and the toxins of venomous animals are of interest in the search for novel anticoagulant leads.

One of the first breakthroughs leading to the current popularity of venoms as a source of biopharmaceutical compounds was the drug captopril, a so-called ‘structure-based design’ drug launched on the market in 1975 [12]. Captopril is derived from a compound found in the venom of the Brazilian lancehead viper *Bothrops jararaca*, and was a highly successful drug used for the treatment for hypertension [13]. Snake venom is of particular interest in coagulation research, with distortion in blood coagulation caused by haemotoxic compounds (e.g., resulting in haemorrhage and/or coagulopathy) [14] being a major toxic effect following envenoming by many species of venomous snakes. A generic and straightforward HTS-based coagulation assay that could be applied in any laboratory would in this case be beneficial.

The research described herein details the optimization, validation and early application of a simple, low-volume HTS coagulation assay, which is colorimetric and plate reader-based and can be used for screening pro- and anticoagulant bioactivities of test compounds or assessing patient clotting capabilities. The assay consists of time-course spectrophotometric measurements of bovine or human plasma incubated with Ca^2+^ and a test compound (optionally other components to assess specific parameters), resulting in kinetic measurements of the clotting profile. In addition to the standard optimization and validation process of the plate reader assay format, the assay was incorporated in a LC–MS analysis workflow with parallel at-line nanofractionation. The nanofractionation approach [15] was taken in order to characterize snake venoms for their pro- and anticoagulation properties. Incorporation of this assay in the complete analytical workflow allows for snake venom component separation by LC followed by parallel high-resolution nanofractionation for subsequent coagulation assessment on the same plate.

## 2. Results

**Figure 1:**
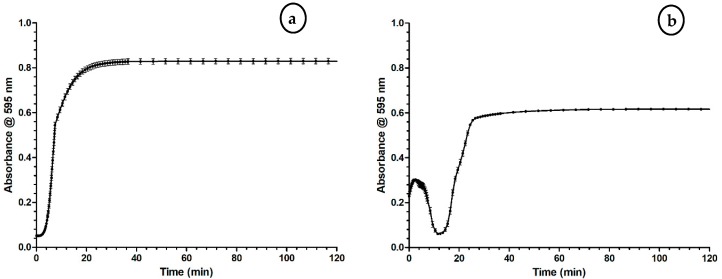
A typical absorbance detection curve upon plate-based coagulation activity monitoring using a plate reader at 595 nm. (**a**) The coagulation activity curve using the approach where plate preparation and measurement in plate reader occurs at room temperature. Coagulation starts and absorbance increases until a plateau (maximum opaque state) has been reached. (**b**) The coagulation activity curve using the approach where plate preparation occurs at 4 °C and the later plate reader measurement at 37 °C. Here, the initial part of the curve shows a fast increase in absorbance caused by temporary formation of condensation on the bottom of the well plate upon placement of the cooled well plate in the plate reader set at a temperature of 37 °C. Both figures show the mean of the coagulation curves (*n* = 32) measured and the error bars represent SEMs (assay conditions: 20 mM CaCl_2_ (20 µL/well) mixed 1:1 with bovine plasma.

The assay procedure in general entails the subsequent addition of a CaCl_2_ solution and citrated plasma to a selected area of a 94- or 384-well plate and colorimetrically measuring formation of coagulation at 595 or 600 nm. Two typical results are shown in Figure 1a,b, representing the coagulation process in vitro. Figure 1a demonstrates the assay preparation at room temperature; Figure 1b demonstrates the assay preparation at 4 °C and plate reader temperature at 37 °C. While both approaches were used here, the primary focus was on the room temperature preparation (Figure 1a) due to greater simplicity of the workflow and the ability to measure the full clotting curve. The 4 °C/37 °C approach allows for manual assay pipetting without initiation of clotting, the drawback being that during the first 10 to 15 min, condensation of water on the well plate bottom results in an initial increase in signal until subsequent evaporation of the condensate (which can be overcome by working in a dry atmosphere).

### 2.1. Optimization of the Coagulation Assay

**Figure 2:**
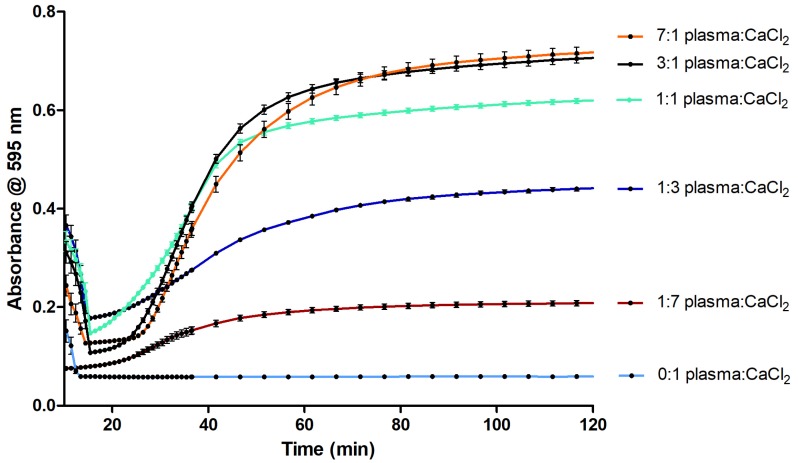
Optimization of the volume ratio of plasma and CaCl_2_. The samples tested ranged from 1:7, 1:3, 1:1, 3:1 and 7:1 final plasma-to-calcium ratios, the total volume per well in all cases was 40 µL and final concentrations of CaCl_2_ were 50 mM. Absorbance measurements were performed as in Section 4.2. Every sample was measured 64 times (four complete columns on a 384-well plate) and the experiment was performed in duplicate. Each curve depicted represents the mean of two columns (32 measurements) of a singular plate with the same plasma-to-CaCl_2_ ratio. This figure shows the mean of the curves measured for each concentration using approach B (i.e., plate preparation at 4 °C and readout at 37 °C) and the error bars represent SEMs.

Different plasma-to-buffer ratios, calcium concentrations and assay volumes were evaluated for assay performance. Figure 2 presents the results of the assay undertaken with varying volume ratios of plasma to CaCl_2_, highlighting that altering this experimental ratio results in major changes in coagulation velocity. The 0:1, 1:3 and 1:7 plasma-to-CaCl_2_ ratios gave a decreased coagulation velocity. The 3:1 and 7:1 plasma-to-CaCl_2_ ratios gave an increased coagulation velocity and higher absorbance compared to the 1:1. However, for all these conditions, the initiation of coagulation was decreased compared to the 1:1 plasma-to-CaCl_2_ ratio.

**Figure 3:**
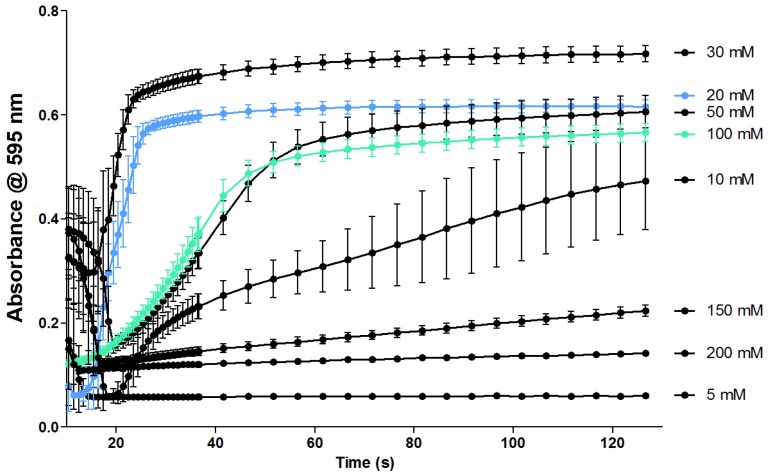
Optimization of the CaCl_2_ concentration. The concentration series of 2, 5, 10, 20, 30, 50, 70, 80, 100, 150 and 200 mM CaCl_2_ (20 µL/well) mixed 1:1 with bovine plasma was tested (final CaCl_2_ concentration thus was: 1, 2.5, 5, 10, 15, 25, 37.5, 40, 50, 75 and 100 mM). Every concentration was measured 48 times (two complete rows on a 284-well plate) and the experiment was performed in triplicate. In the figure, a single experiment is shown and each curve represents the mean of 24 measurements (one concentration per row on one measured 384-well plate) and the error bars represent SEMs. Approach B (i.e., plate preparation at 4 °C and readout at 37 °C) was used to obtain these results.

Altering the concentration of CaCl_2_ used in the assay also produced differences in coagulation velocity (Figure 3). An increase in coagulation velocity was observed from 5 to 30 mM CaCl_2_, with the clotting velocity decreasing at higher calcium concentrations, to the point where coagulation is almost completely inhibited at a concentration of 200 mM CaCl_2_. Finally, the total assay volume was optimized, and the assay was evaluated using both 94- and 384-well plate formats; the results of these experiments are presented in the supporting information (Figures S1 and S2).

### 2.2. Evaluation of the Coagulation Assay with Anti- and Procoagulants

#### 2.2.1. Evaluation of the Coagulation Assay Using Anticoagulant Compounds

Optimized conditions were then chosen and used to demonstrate the coagulation assay with commercially available pharmaceuticals known to inhibit blood coagulation. The compounds tested were Coumadin (warfarin), which depletes the biochemical synthesis of clotting factors, and Acova (Argatroban), a thrombin inhibitor. The results for warfarin, which does not have a significant influence on coagulation in vitro, can be found in the supporting information (Figure S3).

**Figure 4:**
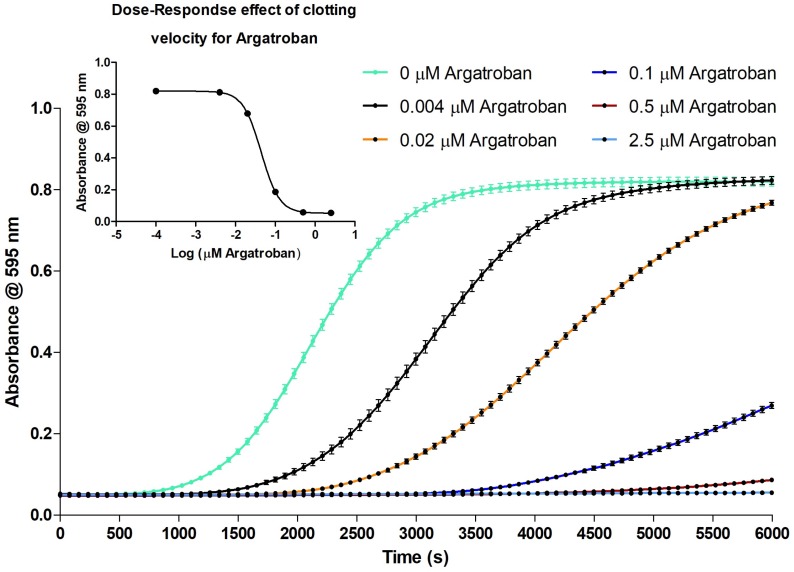
Serial dilution series of Argatroban dissolved in CaCl_2_ mixed 1:1 with bovine plasma (40 µL total volume per well). A 100 µM solution was diluted to a concentration of 25 µM, from which a 1:5 serial dilution series was prepared in 20 mM CaCl_2_. The coagulation assay was performed as described in Section 4.2, replacing the 20 mM CaCl_2_ with Argatroban solutions (in 20 mM CaCl_2_) resulting in Argatroban concentrations of 2.5, 0.5, 0.1, 0.02, 0.004 and 0 µM in the assay. These results were obtained using approach A (i.e., plate preparation and readout at room temperature (21 °C)). The absorbance measurements occurred at 595 nm. Each curve represents the mean of one row (24 measurements) of the duplicate plates with the same concentration of Argatroban. The error bars represent SEMs. The green curve represents the control, where no Argatroban was added.

For Argatroban, the clotting velocity was dose dependent and the inhibitory effect of increasing concentrations on clotting was clearly observed (Figure 4). Furthermore, an EC_50_ curve was plotted from the slopes representing the clotting velocity (Figure 4 insert). The EC_50_ of Argatroban was 43.95 ± 0.005 (nM Argatroban).

#### 2.2.2. Evaluation of the Coagulation Assay Using Russell’s Viper Venom

**Figure 5:**
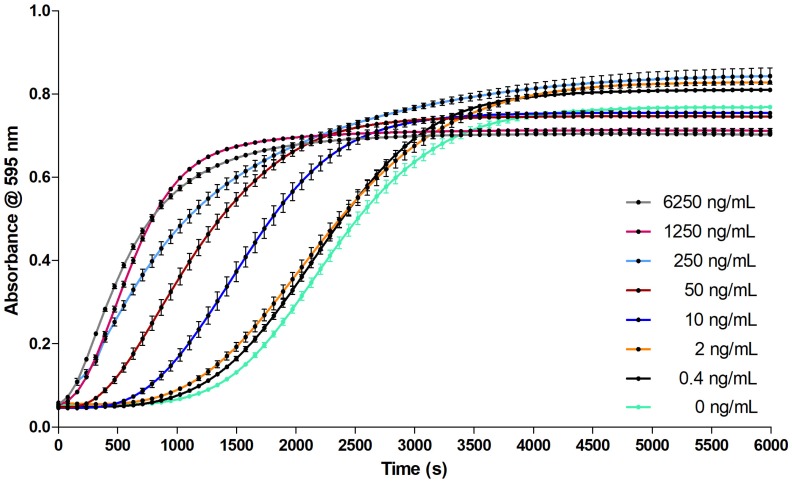
Serial dilution series of Russell’s viper (*Daboia russelii*) venom (RVV) dissolved in CaCl_2_ mixed 1:1 with bovine plasma (40 µL total volume per well). A 5 mg/mL crude venom solution was diluted to a concentration of 0.025 mg/mL, further dilution to 12,500 ng/mL, using a 1:5 serial dilution series prepared in 20 mM CaCl_2_. The assay was performed as described in Section 4.2, but replacing the 20 mM CaCl_2_ solution with the RVV concentration dilution series dissolved in 20 mM CaCl_2_. The final concentrations of RVV tested in the assay were 6250, 1250, 250, 50, 10, 2, 0.4 and 0 ng/mL. Each concentration was measured 48 times (two complete rows on a 384-well plate) and this experiment was performed in duplicate. Each curve represents the mean of one row (24 measurements) of the duplicate plates with the same concentration of RVV. The error bars represent SEMs. The results were obtained using approach A (i.e., plate preparation and readout at room temperature (21 °C)). The absorbance measurements occurred at 595 nm. The green curve represents the control, where no RVV was added.

The coagulation assay was further evaluated with Russell’s viper (*Daboia russelii*) venom (RVV). RVV is known for its coagulopathic activity and composition containing both pro- and anticoagulant venom toxins, and is therefore a suitable venom to validate this assay. These assay evaluation results are presented in Figure 5. The coagulation velocity is dependent on the dose, and the increase in RVV concentration results in an increase in coagulation velocity, visible as an increase in slope steepness of the coagulation curve. However, the steeper coagulation curves show lower maximum absorbance compared to the dilution.

### 2.3. Proof of Concept for the Assessment of Individual Clotting Modulators by Using the Bioassay in Combination with a Nanofractionation LC–MS/MS Workflow

**Figure 6:**
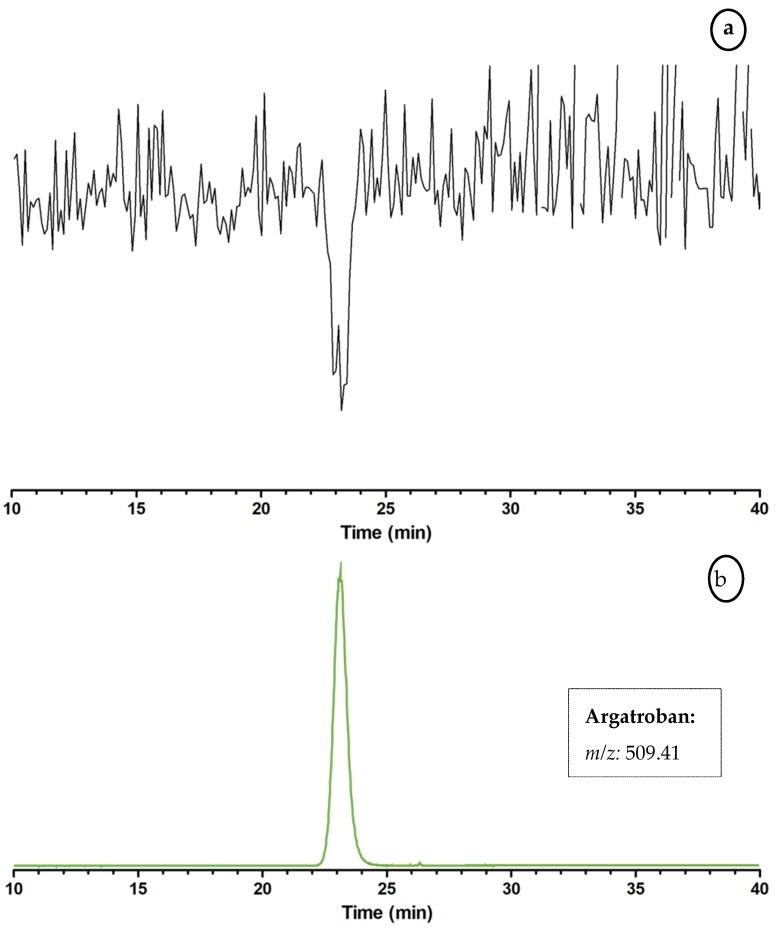
Demonstration of the bioassay implemented in the LC–MS and paralel at-line nanofractionation approach for coagulation activity monitoring using Argatroban. The baseline represents the slopes of the curves per well corresponding to ‘normal’ clotting activity of clotting measured in non-active nanofractions. A negative peak (dip) is observed when wells contain a coagulation inhibitor (i.e., an anticoagulant) since the clotting activity is reduced, resulting in a decrease in the slope of the standard curve. (**a**) Bioactivity chromatogram obtained with the coagulation assay after LC separation of Argatroban, with an injected concentration of 196.6 µM. The coagulation assay was performed by pipetting a freshly prepared 20 mM CaCl_2_ solution and plasma onto the freeze-dried plates containing 6 s nanofractions of the nanofractionated Argatroban. (**b**) The LC–MS result depicted as extracted-ion chromatograms (XICs) of Argatroban, which shows correlation with the inhibiting activity represented as a negative bioactivity peak in (**a**).

The analytical method was first evaluated by using the anticoagulant Argatroban in the LC–MS/bioactivity platform, using Mladic et al.’s nanofractionation strategy [15]. Venom is separated using chromatography followed by a post-column split which directs 10% to MS and the remaining 90% to nanofractionation. Second-range fractions are then collected in serpentine fashion on a 384-well plate (6 s fractions in this study). After nanofractionation, the well plates are vacuum centrifuged for solvent evaporation followed by bioassay pipetting and readout (Experiments were performed to show the LC solvents used have no effect on the coagulation activity [data not shown]). Finally, plotting the measured bioassay responses of the fractions against the fractionation time gives reconstructed bioassay chromatograms. While assessing anticoagulation, the readout of the last measurement was plotted. Figure 6a shows the resulting bioactivity chromatogram. A single negative peak (coagulation inhibition) is visible at 23.1 min. By plotting the extracted-ion chromatogram (XIC) of the [M + H]^+^ ion from Argatroban (*m/z* 509.41) from the parallel obtained LC–MS data (Figure 6b), it is shown that the retention time and shape of the observed peak matches the bioactivity chromatogram.

**Figure 7:**
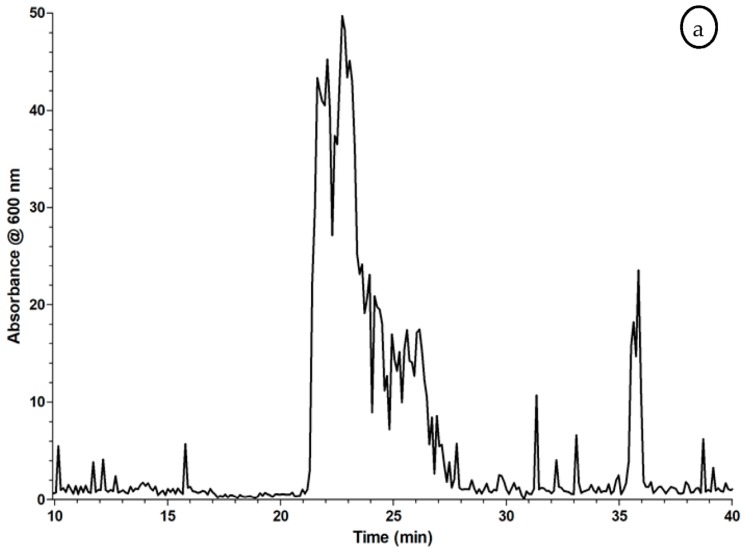

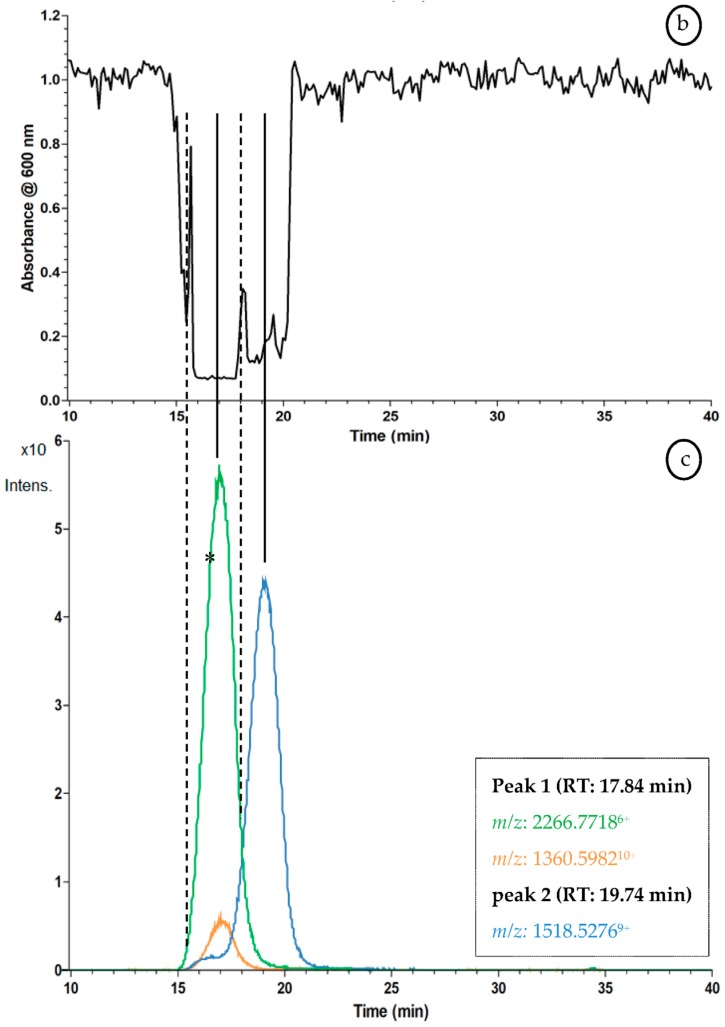
Demonstration of the bioassay implemented in the LC–MS and parallel at-line nanofractionation approach for coagulation activity monitoring using RVV. The baseline represents the slopes of the curves per well corresponding to ‘normal’ clotting activity of clotting measured in non-active nanofractions. A negative peak (dip) is observed when wells contain a coagulation inhibitor (i.e., an anticoagulant) since the clotting activity is reduced, resulting in a decrease in the slope of the standard curve (**b**). When a procoagulant is present (**a**) in adjacent wells, the slope increases relative to the standard curve resulting in a positive peak. (**a**) Bioactivity chromatogram of procoagulation obtained with the coagulation assay after LC separation of RVV, at an injected concentration of 5 mg/mL (50 µL injection volume). (**b**) The corresponding bioactivity chromatogram of anticoagulation. Results shown in (**a**) and (**b**) were derived from the same fractionated plate, but using different ways of data plotting (i.e., plotting slopes and endpoints, respectively). (**c**) The LC–MS result depicted as extracted-ion chromatograms (XICs) of RVV which shows correlation with the inhibiting activities represented as two negative bioactivity peaks in (**b**). The anticoagulation bioactivity chromatogram (**b**) shows a correlation with the data from the parallel MS detection (**c**). No XIC peaks could be correlated with the bioactivity peaks in the procoagulation chromatogram (**a**). For this, nanofractions of interest are to be collected for subsequent proteomics analysis. * shows the retention time of a fraction collected (from a new nanofractionation run) to be used for a de-novo proteomics approach.

The LC–MS/bioactivity platform was then used to screen RVV snake venom for coagulation activity assessment. The resulting bioactivity chromatograms for procoagulation (Figure 7a) and anticoagulation (Figure 7b) were generated as follows: each measurement in the plate reader entails one kinetic loop consisting of 80 readings with interval times of 15 s between every reading. For procoagulant bioactivity chromatograms, the average rate of the curve from reading 1 to 20 was plotted from the plate reader kinetic loop (i.e., 79 to 1577 s) resulting in a procoagulation assessment chromatogram (Figure 7a). For anticoagulation, this procedure cannot be followed, as the signal intensity is much lower and plotting the data in the same manner will result in negative peaks that cannot be distinguished from baseline noise. Therefore, when assessing the inhibition of clotting, another chromatogram is plotted in which the data is normalized using the SkanIt 4.1 software and a single reading of the absorbance of the coagulation curve is plotted, at reading 80 (6232 s) of the measurement (additional information in Section 4.2). The chromatogram for procoagulation (Figure 7a) shows many, partly unresolved, positive peaks eluting between 21 and 27 min, and a sharp peak at 35.3 min. In the 21 to 27 min range, three major peaks can be discerned. The chromatogram for anticoagulation (Figure 7b) shows a smaller peak at 15.5 min and a very broad band between 16 and 20.5 min. Figure 7c shows the LC–MS result depicted as XICs of the accurate masses that were plotted for retention time and peak-shape correlation with the bioactivity peaks. The anticoagulation bioactivity chromatogram correlates to the parallel MS detection data. In Figure 7b, the first eluting broad band (RT: 16 to 20.5 min) covers two peaks in the mass spectral data (Figure 7c). For peak one (RT: 17.8 min), two observed *m/z* values were plotted as XICs that were exactly *m/z* 2266.7718^6+^ and *m/z* 1360.5982^10+^. The accurate masses [M + H]^+^ belonging to these ions were *m/z* 13,594.6308 and *m/z* 13,595.9820, respectively. For peak two (RT: 19.7 min), an accurate mass of *m/z* 1518.5276^9+^ was plotted as XIC that matched the peak in the anticoagulation chromatogram, corresponding to an accurate mass [M + H]^+^ of *m/z* 13,657.7484. To demonstrate protein characterization, a new well plate was nanofractionated with RVV, and the anticoagulant fraction I9, corresponding to retention time 17.2 min (see * in Figure 7c), was processed for de-novo proteomics. More information on this experiment and the proteins identified can be found in the supporting information Table S1.

## 3. Discussion

The colorimetric method employed measures a change in the transmittance of the assay mixture in each well over time, differing from typical methods where a change in color intensity is measured. Coagulation causes increased light scattering and hence a reduced light transmittance, monitored as an increased absorbance using a transparent well plate. Initiation and velocity of coagulation depend on the volume ratio of plasma-to-CaCl_2_ solution, the CaCl_2_ concentration, the oxygen concentration of the ambient air, and the well volume and format (related to the area exposed to air). The HTS assay presented here shows resemblance to a lower-throughput assay variant published by Padula et al. (2017) [16]. Here, the coagulation of citrated plasma was determined in a cuvette using a semi-automated coagulation analyzer by addition of CaCl_2_ in the presence of procoagulant venom, which requires relatively large sample volumes per analysis and has the intrinsic lower-throughput aspect of cuvette-based analysis.

Figure 1a,b demonstrates the different results obtained upon assay preparation. Preparation of the assay must be in close proximity to the plate reader in order to be able to generate Figure 1a successfully. The use of multichannel pipettes, repetition pipettes and/or pipetting robotics are valuable tools to accelerate this process further. If assay preparation and/or transfer of the well plate to the plate reader will take more than 5 min, the plate and all bioassay reagents should be prepared at 4 °C, as lowering the temperature will impede coagulation. In this case, the plate reader is set at 37 °C to induce warming of the plate, increasing the clotting speed, and in turn reducing measurement time (Figure 1b). However, the temperature difference results in water condensation and a decrease in transparency with increased light scattering and hence reduced light transmittance, as is visible in the measurements (Figure 1b). The condensed water quickly evaporates during warming of the plate during measurement.

Although in this scenario condensation prevents the initial part of the clotting curve being measured, a precise assessment of the clotting time can still be made as the formed condensate evaporates prior to the majority of coagulation occurring. Condensation formation can be prevented by working in a dry plate reader atmosphere. In both approaches the relative coagulation activity is determined by the slope of the curves, with the steeper the slope the faster maximum absorbance is reached (i.e., the sooner coagulation is completed) and vice versa. When visually inspecting the wells after measurement, clots can be observed, and are easily differentiated from wells where no clots have been formed (Figure S4 in the supporting information).

### 3.1. Optimization of the Coagulation Assay

The optimal volume ratio of plasma-to-CaCl_2_ solution was found to be 1:1 due to the minimal decrease of clotting initiation and the velocity, the steepness of the curve and absorbance level (Figure 2). Subsequently, the 20 mM concentration of CaCl_2_ (final assay concentration of 10 mM) was considered optimal in terms of coagulation velocity, compatibility with assay preparation time, and time needed for placement in the plate reader to initiate the readout (Figure 3). This is in line with the literature where optimal CaCl_2_ concentrations between 10 and 20 mM are considered [17,18,19]. At a concentration of 5 mM CaCl_2_ (final assay concentration of 2.5 mM), no coagulation was observed; this concentration is therefore insufficient to titrate the citrate. The minimum concentration of CaCl_2_ for which clot formation is observed is 10 mM (final assay concentration of 5 mM). The large error bars highlight that the velocity of coagulation varies significantly between wells, implying that this concentration of Ca^2+^ is only just sufficient to titrate the citrate. However, this does not leave a significant excess of Ca^2+^ ions required to initiate the coagulation cascade in a repeatable manner. The 30 mM CaCl_2_ (final assay concentration of 15 mM) gives the fastest coagulation velocity, however, the absorbance measured during the first measurement cycle is already significantly increased compared to all other coagulation conditions tested. This implies that clot formation had already started prior to the placement of the measurement plate in the plate reader. Further increases in concentration result in further decreases in coagulation velocity. The optimal final assay volume was found to be 40 µL in the plasma-to-CaCl_2_ solution ratio of 1:1 for a 384-well plate, and upon final assay volume adjustment to 80 µL, the assay equally worked for a 96-well plate (Figures S1 and S2 in the supporting information).

### 3.2. Evaluation of the Coagulation Assay with Anti- and Procoagulants

#### 3.2.1. Evaluation of the Coagulation Assay Using Anticoagulant Compounds

The coagulation assay was evaluated with commercially available pharmaceuticals known to inhibit blood coagulation to confirm the suitability of the in-vitro coagulation assay as a proxy for the in-vivo mechanism of blood coagulation. Argatroban directly inhibits the serine protease thrombin thereby preventing thrombin from converting fibrinogen into fibrin clots and preventing platelet aggregation [20,21]. The effect of such pharmaceuticals on coagulation was tested by measurement of serial dilutions of both warfarin (detailed in supporting information) and Argatroban, to determine the EC_50_. Different studies have been published determining the IC_50_ values for Argatroban in colorimetric experiments using chromogenic substrates [22,23,24] and also using standard PT coagulation tests using different PT reagents [25]. In this study, however, the effect of Argatroban on clotting is studied colorimetrically, without the addition of any tracer substrate, ligand and reagents, resulting in the determination of the effective concentration 50 (EC_50_) of Argatroban towards blood coagulation directly, rather than an IC_50_. Here it can be seen that an increase of the concentration of Argatroban gives a reproducible decrease of the coagulation velocity, as observed by a decrease in the steepness of the coagulation curve. Within this serial dilution, full coagulation inhibition is achieved at an Argatroban concentration of 2.5 µM (Figure 4). The EC_50_ was determined to be 43.95 ± 0.005 (nM Argatroban). This data confirmed that the clotting velocity is dose dependent and the inhibitory effect of increasing concentrations of Argatroban on clotting is clearly observed (Figure 4).

#### 3.2.2. Evaluation of the Coagulation Assay Using Russell’s Viper Venom

Snake venoms are complex mixtures of varying bioactive proteinaceous components, which can be enzymatic or nonenzymatic, and are typically referred to as toxins [26]. Venoms, in particular certain snake venoms, are known to contain a multitude of compounds that influence coagulation [14]. These compounds, mostly proteins, can be classified into two major groups, namely procoagulants and anticoagulants, as they either prolong or reduce the coagulation time, respectively [27,28]. Most procoagulant snake venom toxins can be classified as metalloproteinases or serine proteases and they induce consumption coagulopathy following snakebite as the result of these toxins continuously activating various components of the clotting cascade [14,29]. One of the main targets for procoagulant venom toxins is prothrombin. Toxins that activate this clotting factor have been classified into four major groups (A–D) [30], which are comprised either of metalloproteinases that are structurally unrelated to coagulation factors (A and B) or serine proteases that are structurally similar to vertebrate coagulation factors (group C and D) [30]. Other procoagulant toxins work by activating Factor X and/or Factor V or by converting fibrinogen into fibrin.

In addition, many snake venoms contain anticoagulant toxins (often in conjunction with procoagulant toxins); these are also highly variable and include enzymatic and nonenzymatic proteins, for example, phospholipase A_2_ (PLA_2_) and C-type lectins, respectively, although several other toxin types can also be involved [14]. In this study, the well-characterized RVV was used to measure the clotting activity of its coagulopathic compounds. RVV is a suitable venom to validate this assay due to its coagulopathic activity and composition containing both pro- and anticoagulant venom toxins. RVV is known to contain metalloproteinases as well as serine proteinases, contributing to coagulation distortion [31,32]. Furthermore, RVV has been found to contain Factor V and Factor X activators (i.e., metalloproteinases and serine proteases) that cause severe coagulopathy and bleeding disturbances [28]. In addition, it also contains a high concentration of PLA_2_s.

Increasing the RVV concentration in the assay resulted in an increase in coagulation velocity (Figure 5). When using snake venom to induce coagulopathic activity in this assay, the steeper coagulation curves showed lower maximum absorbance. This may be due to the density of the formed clot and fibrin crosslinking, which probably is different during lower coagulation velocities. The data suggests that RVV only has coagulopathic enzymes that activate the endogenous clotting cascade (to an enzymatic maximum clotting velocity dependent on the prothrombin concentration in the plasma) and do not have independently acting clotting factors mimicking endogenous clotting factors. Figure 5 demonstrates this suggestion since maximum coagulation velocity was reached and failed to increase further by higher RVV assay concentrations (6250 and 1250 ng/mL show no substantial difference in the slope of the curve).

### 3.3. Proof of Concept for the Assessment of Individual Clotting Modulators by Using the Bioassay in Combination with a Nanofractionation LC–MS/MS Workflow

The developed assay was then applied for identification of individual peptides and enzymes with haemotoxic properties in venoms. The coagulation assay was implemented in an analytical approach, described in Section 4.3. RVV venom consists of many different protein components that vary extensively in terms of their molecular weight and functional activity [33,34]. RVV venom gives a characteristic bioactivity profile reflecting the bioactivity of the eluted compounds (Figure 7a,b), suggesting the presence of at least three major procoagulating compounds and at least two components in the venom with anticoagulant activity. However, this venom most likely contains many more masked co-eluting peaks. It should be noted that the LC chromatographic conditions applied could potentially cause partial and/or full denaturation of certain venom enzymes, which obviously can affect the resulting activity of the components present in the sample. The two bioactivity chromatograms represent procoagulant and anticoagulant bioactivity, respectively (explained in Section 2.3 and Section 4.2). Figure S5 (supporting information) presents an example where combining the two bioactivity chromatograms of procoagulation and anticoagulation is attempted.

Figure 7c presents the parallel-obtained MS data. Considering the masses found (Figure 7c), the first two peaks are suggested to be phospholipases A_2_ (PLA_2_), as their MW is around the 13.6 kDa, the generic molecular weight of PLA_2_s [35]. To confirm the identity of the correlated peaks found in the bioassay and MS, a de-novo sequencing proteomics approach was pursued. This encompassed nanofractionation of RVV, followed by on-plate tryptic digestion and nano LC–MS/MS analysis towards protein identification. The fraction (I9) corresponding to retention time of 17.2 min, which includes the first eluting peak from the anticoagulant screening (depicted by * in Figure 7c), was picked for demonstration of this approach. After tryptic digestion procedure of this well, de-novo sequencing and database search, several hits related to snake venom PLA_2_s were found (see supporting information Table S1). In brief, from this measurement, two anticoagulant PLA_2_s were identified. This data represents a proof of concept, and further details of the proteomic identification of bioactive venom toxins exceed the scope of this paper.

### 3.4. Summary & Research Applications

This research optimized, validated and demonstrated the proof of concept of a HTS coagulation assay. Most routine coagulation assays are arduous, involving the addition of special reagents, have narrow specificity, or require specialized detection equipment. The value of this assay is based on its simplicity, low sample volume requirements, cost-effectiveness, and no need of special reagents. The general assay procedure entails the addition of 20 µL CaCl_2_ and 20 µL citrated plasma to a selected area of a 384-well plate and colorimetrically measuring coagulation at 595 or 600 nm. Close proximity to the plate reader and the use of multichannel pipettes, repetition pipettes and/or pipetting robotics are valuable tools to improve the quality of the results. Not only does this assay monitor general coagulation activity, it shows distortion in coagulation activity towards coagulopathic compounds such as known biopharmaceuticals (Argatroban) and peptides and enzymes with haemotoxic properties in snake venoms. This suggests its suitability as an asset for haemotology and coagulation research in addition to the biopharmaceutical industry for detecting compounds with coagulant effects. Furthermore, this assay may represent a valuable new tool to study one of the most devastating effects of snakebites: venom-induced coagulopathy.

Incorporation of the assay in the LC–MS analysis workflow with parallel at-line nanofractionation has allowed snake venom component separation and screening of components influencing blood coagulation. The parallel-assessed MS-based identification of coagulopathic RVV components allows for proof-of-concept demonstration. Accurate mass data was provided for the most active anticoagulants, where simultaneously the coagulation bioactivity chromatogram could be plotted and can serve as a fingerprint chromatogram for studying venom-induced coagulation in general.

Furthermore, the incorporation of the assay in a standard plate-based and plate reader setup makes it universally suitable to different types of laboratories, enabling high-throughput assessments. Further prospects for this assay, not shown in this paper, encompass its potential use in clinical diagnostics. The standardized coagulation tests, such as the TT, PT and aPTT, could possibly be adapted to the presented assay format allowing these tests to be performed in a universally applicable and high-throughput setup using only low sample volumes. Combining plasma from human patients with a panel of selective compounds known to stimulate or inhibit different components of the clotting cascade would present clinicians with a valuable high-throughput tool to rapidly identify the underlying physiological deficiencies responsible for causing blood clotting disorders.

## 4. Materials and Methods

### 4.1. Chemical and Biological Reagents

Water was purified with a Milli-Q Plus system (Millipore, Amsterdam, The Netherlands). DMSO was supplied by Riedel-de-Haën (Zwijndrecht, The Netherlands). Acetonitrile (ACN, Concord, NC, USA; ULC/MS grade) and formic acid (FA) were obtained from Biosolve (Valkenswaard, The Netherlands). All salts, such as CaCl_2_, used for buffer preparation were of analytical grade and purchased from standard suppliers (Merck, Kenilworth, UK; Fluka, Bucharest, Romania; or Sigma-Aldrich, Darmstadt, Germany). Micro-90^®^ concentrated cleaning solution was supplied by Sigma-Aldrich (Darmstadt, Germany). Lyophilised Russell’s viper (*Daboia russelii*; origin Sri Lanka) snake venom (RVV) was provided by the Alistair Reid Venom Research Unit Herpetarium (Liverpool School of Tropical Medicine, UK) and stored long-term at 4 °C. Stock solutions of crude venoms (5.0 ± 0.1 mg/mL) were prepared in water prior to analysis and stored at −80 °C. The 100 µM stock solution of Argatroban was made in DMSO and kept at 4 °C. Bovine plasma (500 mL bottle) was purchased from Biowest (Nuaillé, France). The plasma was aseptically collected and sodium citrated (amount not specified by supplier) bovine whole plasma and was tested for sterility and endotoxicity by the supplier. The plasma was defrosted in a water bath at 37 °C, centrifuged, and aliquoted (approximately 11 mL in 15 mL Costar tubes) and kept at −80 °C.

### 4.2. Coagulation Activity Assay

The assay was run by measuring coagulation dynamically (in-time) in plate reader format (most experiments performed in 384-well plate), as an increase in absorbance (more precisely a decrease in transmittance) at a wavelength of 595 or 600 nm during fibrinous clot formation. The addition of an excess of CaCl_2_ solution to titrate away the citrate in citrated plasma initiated clot formation. Different calcium concentrations, assay volumes and plasma-to-buffer ratios were evaluated for assay performance. Optimized conditions were then chosen and used to demonstrate the assay with anticoagulants (drugs) and procoagulants (Russell’s viper (*Daboia russelii*) venom (RVV)).

Assay preparation was performed at room temperature (unless stated otherwise). As coagulation is initiated by the mixing of plasma with the calcium solution, the assay preparation must be performed rapidly, and the plate reader measurement must be initiated, ideally within 5 min. For measurements, a fresh working solution of 20 mM CaCl_2_ was prepared (2 weeks expiration time when kept at 4 °C) and frozen aliquots of plasma, either citrated bovine plasma or pooled human plasma, were rapidly defrosted in a water bath (37 °C). Within an hour of defrosting, the plasma was used. Before robotic pipetting (completed with a well plate ThermoScientific™ Multidrop™ 384 Labsystems (see supporting information S1 for the cleaning procedure)), the plasma was centrifuged for 20–30 s at 1400 rpm with an Eppendorf 5810 R centrifuge. The general assay procedure was performed by robotically pipetting CaCl_2_ (20 µL/well) followed by plasma (20 µL/well) over a selected area of a 384- well plate.

The prepared plate, containing 40 µL CaCl_2_ solution and plasma (1:1) per well, was always placed in the plate reader within 5 min after assay mixture pipetting (unless stated otherwise). The plate reader temperature was set at room temperature (unless stated otherwise). The absorbance was measured at a wavelength of 595 or 600 nm with a Thermo Fisher Scientific Laboratory Varioskan™ LUX Multimode Microplate Reader using SkanIt 4.1. Measurements were performed in one kinetic loop consisting of up to 80 readings, each 80 s long (65 s + 15 s interval time), resulting in a total measurement time of 6400 s per kinetic loop. Three data-processing options in the SkanIt 4.1—(1) single reading, (2) slope of a reading range, and (3) average rate in time per well—were used to determine both procoagulation and anticoagulation activity by processing the coagulation curves (further details in Section 2 and Section 3). The bioassay data is presented as a bioassay chromatogram when used for snake venom screening (Section 2.3). When there is a significant difference in signal intensity between anti- and procoagulation, it should be noted that both procoagulation and anticoagulation activities cannot be monitored in the same manner (details in Section 2.3).

### 4.3. Instrumental Setup

Separation of mixtures for post-column assessment with the coagulation assay was completed by LC with high-resolution nanofractionation, following Mladic et al. [15]. Samples were injected with a Shimadzu SIL-20AC autosampler and LC separation was performed with an LC system controlled via Shimadzu Lab Solutions software. The gradient was set using two Shimadzu LC-20AD pumps (A and B) at a total flow rate of 0.5 mL/min. A 250 × 4.6 mm ID analytical column was packed with Xbridge^TM^ BEH300 reversed-phase C18 material (3.5 µm) and maintained at 37 °C in a Shimadzu CTD-30A column oven. The gradient was set using two Shimadzu LC-20AD pumps (A and B) at a total flow rate of 0.5 mL/min. Mobile phase A was water–ACN–FA (98:2:0.1, v/v/v) and mobile phase B was water–ACN–FA (2:98:0.1, v/v/v). The following gradient was used: 0% to 50% B (20 min), 50% to 90% B (4 min), 90% B (5 min), 90% to 0% B (1 min), 0% B (10 min). The column eluate was split in a 1:9 ratio using a low-dead-volume flow splitter. The smaller flow (0.05 mL/min) led to a high-resolution time-of-flight (TOF) mass spectrometer for selective detection and compound identification. The larger eluate flow was fractionated (6 s/well) onto transparent 384-well plates (Greiner Bio One, Alphen aan den Rijn, the Netherlands) employing a system controlled by Ariadne software. After nanofractionation the plates were overnight vacuum centrifuged to dryness at room temperature using a Christ Rotational Vacuum Concentrator RVC 2-33 CD plus (Salm en Kipp, Breukelen, the Netherlands), using a cooling trap at −80 °C. The plates were always stored at −80 °C. By plotting the measured bioassay responses of the fractions against the fractionation time, bioassay chromatograms were reconstructed.

A MaXis II QTOF mass spectrometer (Bruker Daltonics, Bremen, Germany), equipped with an electrospray ionization source (ESI) and operated in positive ion mode, was used. The parameters of the ESI source were: source temperature, 180 °C; desolvation temperature, 180 °C; capillary voltage, 4500 V; gas flow, 4 L/min. The monitored mass range was *m/z* 50–2000 range with a data-sampling time of 1 s. The parallel-acquired MS chromatogram was used for correlation of bioactivity peaks to compound mass spectra.

## Supplementary Materials

The supporting information related to this article can be found in “*Supplementary Materials: Multipurpose HTS coagulation analysis: assay development and assessment of coagulopathic snake venoms*”. The following are available online at http://www.mdpi.com/2072-6651/9/12/382/s1, Figure S1: Optimization of the final total assay volume in 384-well plate, Figure S2: Optimization of the final total assay volume in 96-well plate, Figure S3: Serial dilution series of warfarin dissolved in CaCl_2_ mixed 1:1 with bovine plasma (40 µL total volume per well), Figure S4: Demonstration of the bioassay implemented in the LC–MS and parallel at-line nanofractionation approach for coagulation activity monitoring using RVV, with an injected concentration of 5 mg/mL, Figure S5: Demonstration of the bioassay implemented in the LC–MS and parallel at-line nanofractionation approach for coagulation activity monitoring using RVV, Table S1: Proteomics data obtained after fractionation, tryptic digestion, de-novo sequencing and database search.
