# Supplementary material for: Multipurpose HTS Coagulation Analysis: Assay Development and Assessment of Coagulopathic Snake Venoms
Source: Toxins (Basel). 2017 Nov 25;9(12):382. doi: 10.3390/toxins9120382 (PMC5744102; doi:10.3390/toxins9120382)
Supplement: Supplementary file 1 [file toxins-09-00382-s001.docx]

Supplementary Materials: Multipurpose HTS Coagulation Analysis: Assay Development and Assessment of Coagulopathic Snake Venoms

Kristina B. M. Still, Randjana S. S. Nandlal, Julien Slagboom, Govert W. Somsen, Nicholas R. Casewell and Jeroen Kool

S1. Cleaning procedure ThermoScientific™ Multidrop™ 384 Labsystems pipetting robot

Prior to pipetting the assay on the well plate, the ThermoScientific™ Multidrop™ 384 Labsystems pipetting robot was always cleaned by subsequently flushing the system with 20% ethanol, Millipore grade water, cleaning solution [2% Micro-90® concentrated cleaning solution] and finally Millipore grade water. After these washing steps, the pipetting robot was flushed with CaCl_2_ solution (usually 20 mM) at room temperature, after which this solution (20 µL per well) was directly pipetted onto a 384 well plate at room temperature. This was followed by flushing the pipetting robot, in order to prevent clotting in the robotics tubing, with a sequence of Millipore grade water, cleaning solution and Millipore grade water. The robotics tubing of the pipetting robot was then completely filled with plasma at room temperature after which the plasma (20 µL per well) was pipetted into the CaCl_2_ filled wells. The plate was always placed in the plate reader within 5 minutes after assay mixture pipetting (unless stated otherwise). The plate reader temperature was set at room temperature (unless stated otherwise). The absorbance was measured with a Thermo Fisher Scientific Laboratory Varioskan™ LUX Multimode Microplate Reader using the SkanIt software 4.1 at a wavelength of 595 nm.

S2. Optimization of assay volume on a 384 well plate


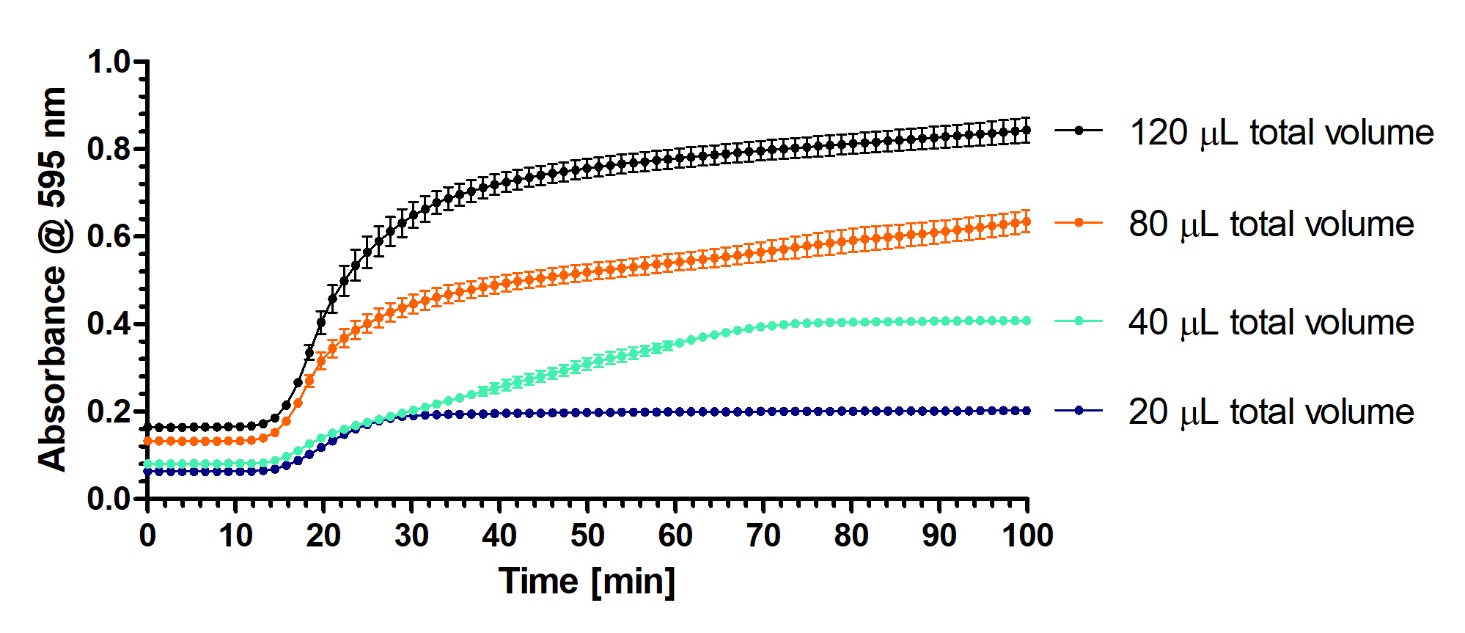


**Figure S1.** Optimization of the final total assay volume in 384-well plate. The different volumes tested were 20, 40, 80 and 120 µL final volume in a plasma to CaCl_2_ ratio of 1:1. This figure shows the mean readings measured for each sample using approach A (i.e. plate preparation at room temperature (21 ℃)). In the figure, a single experiment is shown and each curve represents the mean of 8 measurements (on one measured 384 well plate) the error bars represent SEMs. The optimal final assay volume was found to be 40 µL in the plasma to CaCl_2_ solution (20 mM) ratio of 1:1 for a 384 well plate, due to the combination of steepness of the curve, absorbance level and most reproducible results.

S3. Coagulation assay on a 96 well plate


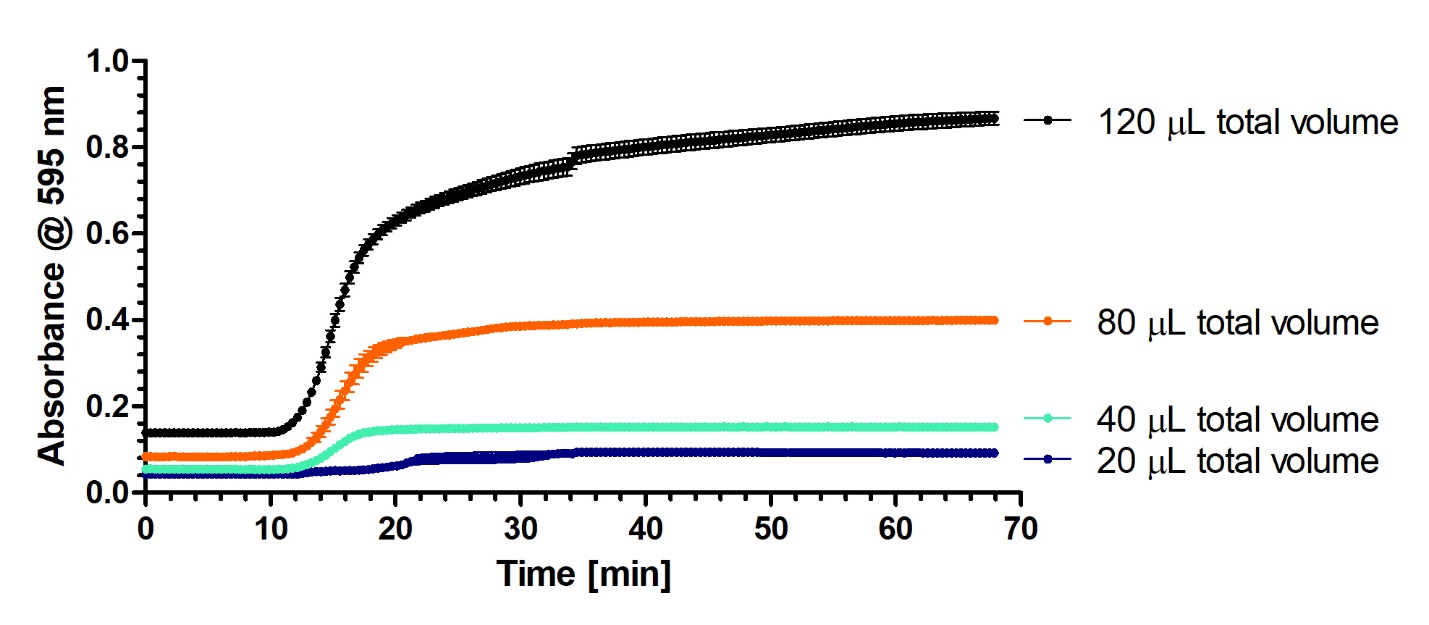


**Figure S2.** Optimization of the final total assay volume in 96-well plate. The different volumes tested were 20, 40, 80 and 120 µL final volume in a plasma to CaCl_2_ ratio of 1:1. This figure shows the mean readings measured for each sample using approach A (i.e. plate preparation at room temperature (21 ℃)). In the figure, a single experiment is shown and each curve represents the mean of 8 measurements (on one measured 384 well plate) the error bars represent SEMs. The final assay volume had to be adjusted in comparison to the 384 well plate, in the 96 well plate, the 40 µL does not fill the individual wells enough and therefore does not give optimal results. For the 96 well plate, the optimal final assay volume was found to be 80 µL in the plasma to CaCl_2_ solution (20 mM) ratio of 1:1.

S4. Serial dilution of Warfarin

Warfarin, a commonly used anticoagulant drug, interrupts the synthesis of Vitamin K which plays a key role in the production of the coagulation factors II, VII, IX and X, and the anticoagulant proteins C and S. Warfarin, administered *in vivo* results in inhibition of the coagulation cascade by lowering expression levels of coagulation factors [1][2]. Consequently, Warfarin acts as a negative control as it does not have any influence on the *in vitro* clotting assay since all the necessary clotting factors are already present in the plasma used. Argatroban (results in the main text) acts as a positive control since it does influence the in vitro assay by directly blocking thrombin. Warfarin indeed, has no influence on coagulation in the assay (all different curves within the serial dilution show great overlap with the curve of the blank), as shown in Figure S3.


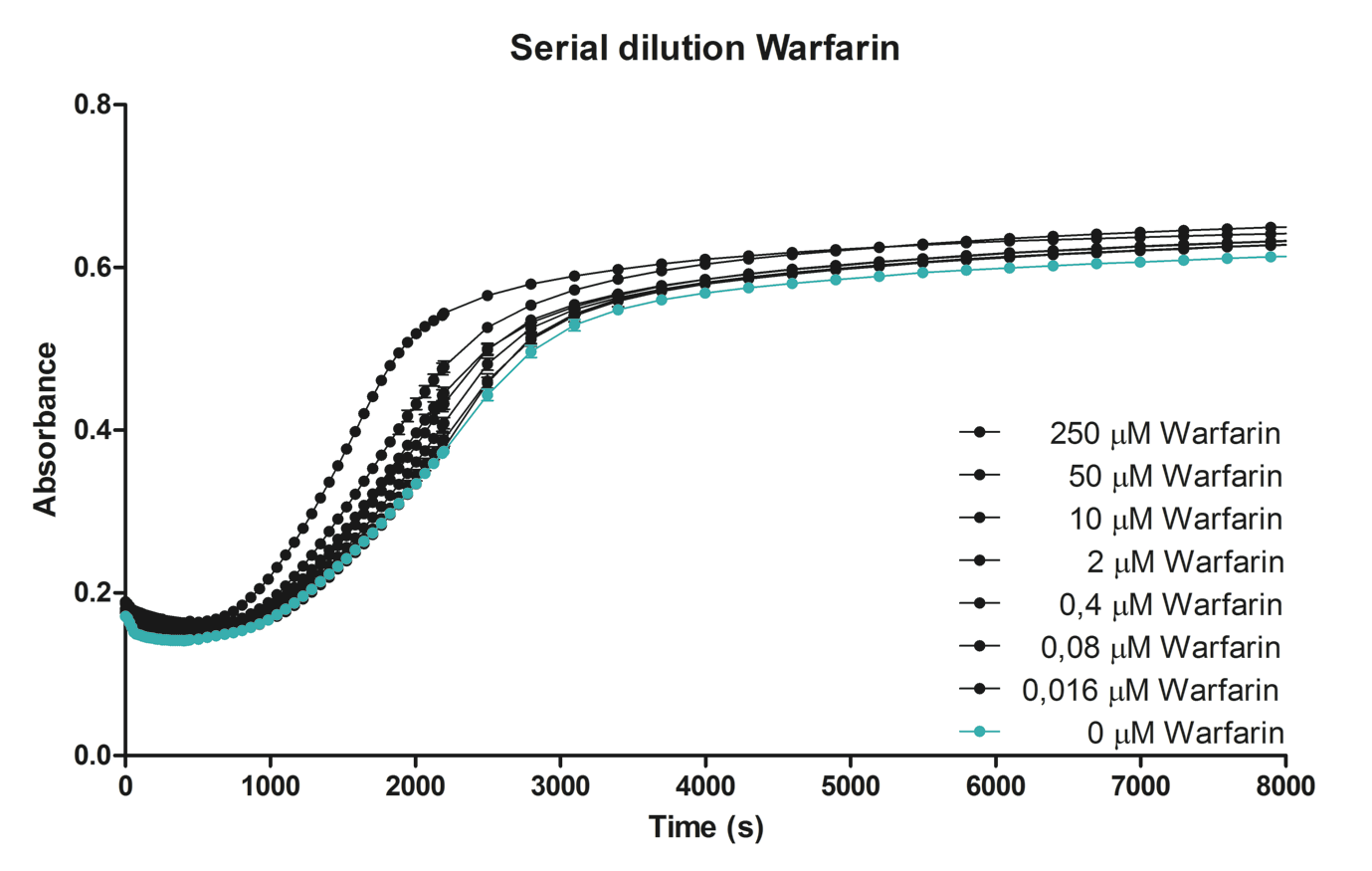


**Figure S3.** Serial dilution series of Warfarin dissolved in CaCl_2_ mixed 1:1 with bovine plasma (40 µL total volume per well). These results were obtained using approach B (i.e. plate preparation at 4℃ and readout at 37℃). The absorbance measurements occurred at 595 nm. Final assay concentrations are depicted in the figure. Each curve represents the mean of one row (24 measurements) of the duplicate plates with the same concentration of Warfarin. The error bars represent SEM. The green curve represents the control, where no Warfarin was added. Minimal effects on clotting are observed, an increase in concentration of Warfarin (anti-coagulant) does not give a decreased coagulation velocity compared to the control coagulation result. This is in accordance to the assay principle as Warfarin interferes with the Vitamin K synthesis and not directly interacts with clotting factors.

S5. Picture of coagulation assay on a 384 well plate.


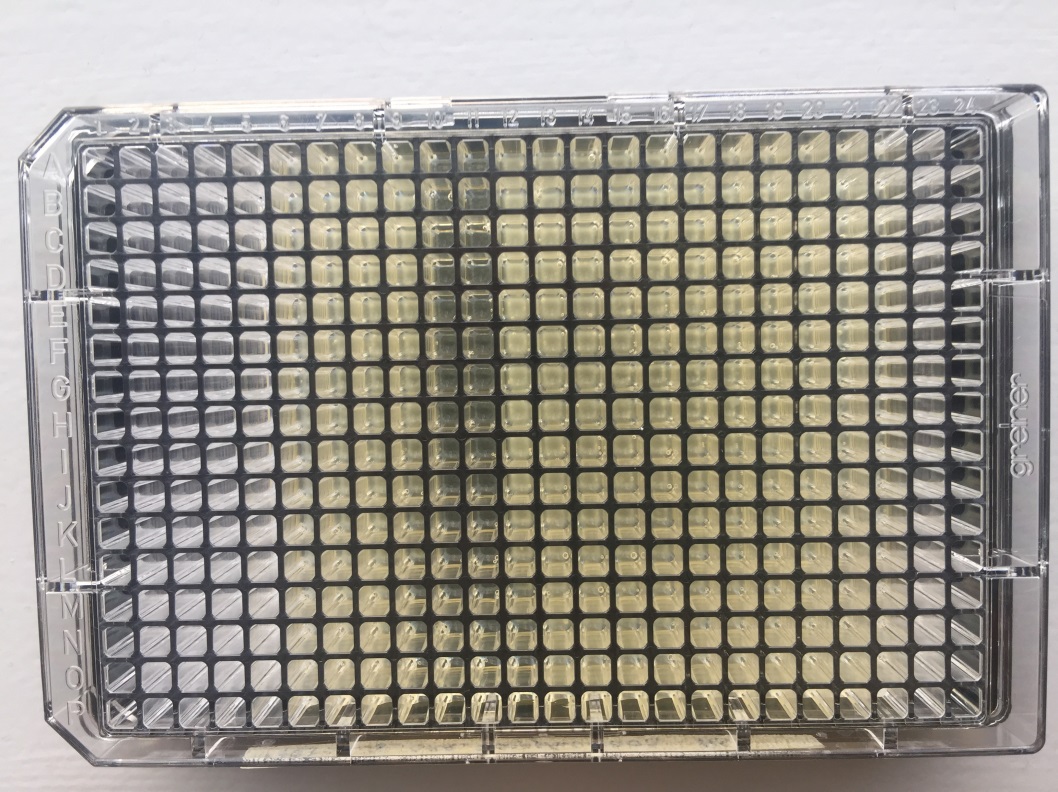


**Inhibition**

**1 2 3 4 5 6 7 8 9 10 11 12 13 14 15 16 17 18 19 20 21 22 23 24**

**A**

**B**

**C**

**D**

**E**

**F**

**G**

**H**

**I**

**J**

**K**

**L**

**M**

**N**

**O**

**P**

**Figure S4.** Demonstration of the bioassay implemented in the LC-MS and parallel at-line nanofractionation approach for coagulation activity monitoring using RVV. The figure shows a picture of the plate which contains the coagulation assay after LC-separation of RVV, with an injected concentration of 5 mg/mL. Columns 1-5 and 23-24 do not contain assay mixture and are therefore transparent. Columns 6-22 do contain assay mixture (40 µL CaCl_2_ and plasma in the ratio 1:1). All wells show light yellow shade; coagulation has occurred. Column 10 and 11 are transparent, these wells show inhibited coagulation as they contain inhibitory components present in the venom. This figure shows that coagulation and anti-coagulation is well differentiable even with the human eye.

S6. Combined pro-, and anti-coagulation bioactivity chromatogram
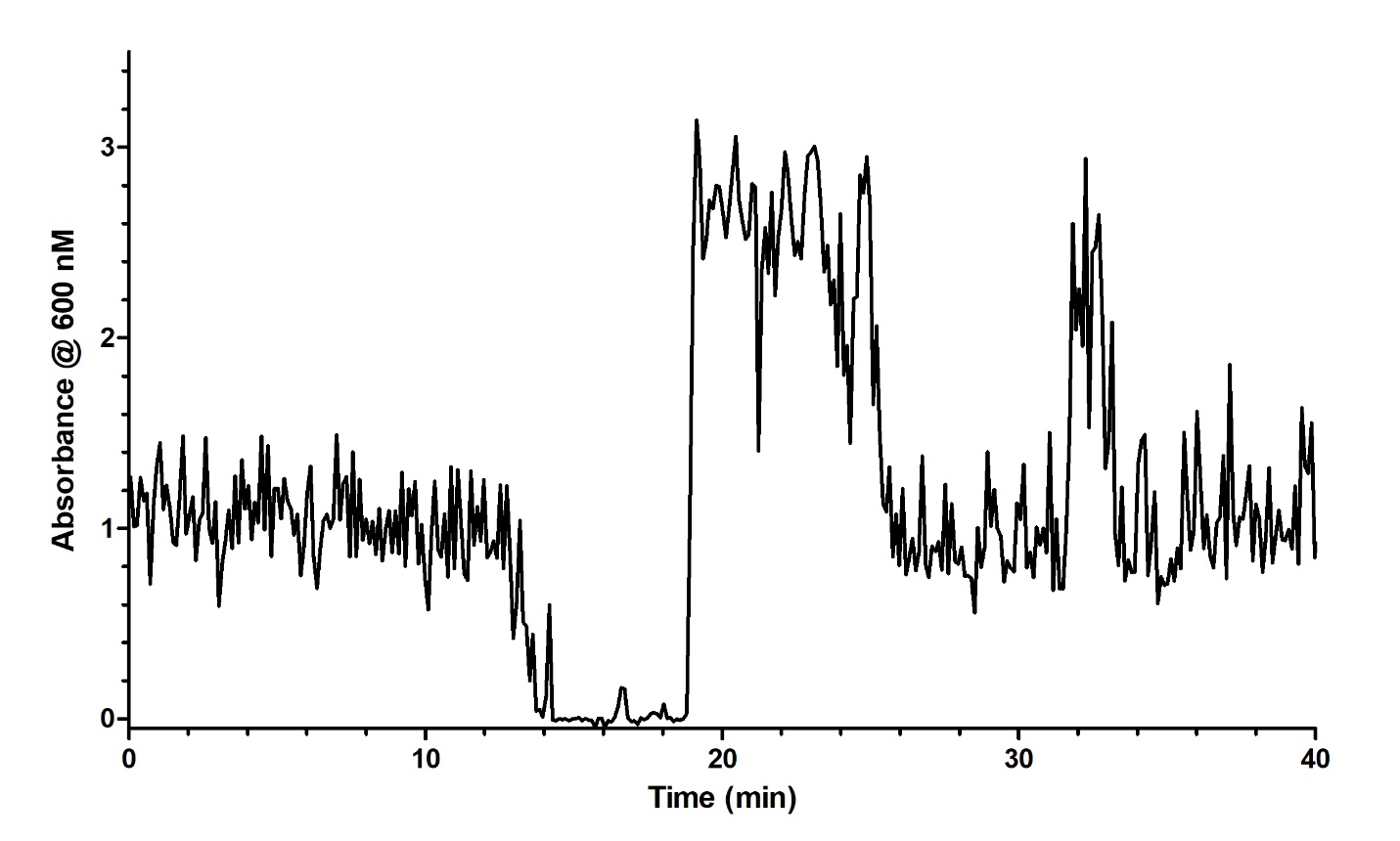


**Figure S5.** Demonstration of the bioassay implemented in the LC-MS and parallel at-line nanofractionation approach for coagulation activity monitoring using RVV. Bioactivity chromatograms of both pro-coagulation and anti-coagulation obtained with the coagulation assay after LC-separation of RVV (injected concentration 5 mg/mL). The coagulation assay was performed by pipetting a freshly prepared 20 mM CaCl_2_ solution and plasma on the freeze-dried plates containing 6s nanofractions of the nanofractionated RVV. The slope from a curve from reading 1 to reading 30 is plotted from the plate reader kinetic loop (i.e. 78.88 seconds to 2,366.40 seconds) resulting in a combined bioactivity chromatogram. As can be seen, the pro-coagulant activity is highly reduced compared to the procedure used to obtain the pro-coagulant activity chromatogram shown in the main text.

S7. Proteomics approach

Table S1 gives the results after proteomics data analysis of fraction I9 (depicted with an * in Figure 7c (main text)).

**Table S1.** Proteomics data obtained after fractionation, tryptic digestion, de novo sequencing and database search. Fraction I9 (RT: 17.2 min) results in several hits corresponding to snake venom PLA2s. OS = originating species.

| **Well I9** | **Compound information** | |
| --- | --- | --- |
|  | *PA2BS_DABSI* | Basic phospholipase A2 DsM-S1- OS=*Daboia siamensis*  Protein score: 882, Sequence coverage: 78% |
|  | *PA2B5_DABRRI* | Basic phospholipase A2 VRV-OL-V- OS=*Daboia russelii*  Protein score: 445, sequence coverage: 41% |
|  | *NGFV_DABRR* | Venom nerve growth factor- OS=*Daboia russelii*  Protein score: 199, sequence coverage: 47% |

**References Supplementary Information**

1. Merli GJ, Fink J. Vitamin K and Thrombosis. *Vitam. Horm*. **2008**, *78*, 265-279, doi:10.1016/S0083-6729(07)00013-1.

2. Lomonaco T, Ghimenti S, Piga I, et al. Monitoring of warfarin therapy: Preliminary results from a longitudinal pilot study. *Microchem. J.* **2017**, doi:10.1016/j.microc.2017.02.010.
